# Eternal Vigilance

**Published:** 1900-06-09

**Authors:** 


### "ETERNAL VIGILANCE."

The price of liberty, it is said, is eternal vigilance; and so, apparently, is the price of asepsis. It is unfortunately the fact that when asepsis breaks down it is nearly always in consequence of undue trustfulness. The surgeon who does aseptic surgery in the modern sense must either be himself an enthusiast, or he must have chanced upon a group of assistants blessed with an enthusiasm as rare as it is delightful. But, in the latter case, unless eternal vigilance is exercised a day of reckoning comes. A weak-kneed member finds entry into the band of the aseptic brethren, and a routine, which is perfection when run with zeal, breaks down when even one person fails in carrying it out with rigid completeness. There is no doubt that the strong point in the rough and ready use of chemical antiseptics lies in the fact that the surgeon uses them himself at the very moment of operation; while the weak point of asepsis lies in the number of persons in whose well-disciplined enthusiasm confidence must be placed. The porters and others to whom the working of large steam sterilisers is sometimes entrusted are, no doubt, very good fellows, but they may not always quite recognise the aim of their performances, and being human, they may sometimes be in a hurry. Hence we can well believe the story of the anxious house surgeon who, seeing that the progress of the cases was not entirely "uneventful," as the saying is, sought out the cause of the catastrophe by placing a tell-tale piece of wax candle among some pads before sending them to be sterilised. The tale goes that although the pads were returned "sterilised" according to the proper formula, the wax was unchanged, having passed unmelted through the ordeal by superheated steam! We will not go so far as to say that a surgeon who wants to do aseptic work must put faith in himself alone, but we will assert that the price of asepsis is eternal vigilance, and that by the exercise of such vigilance alone can it be maintained.

